# Comparison of the Clinico-Demographic Profile of Patients Admitted to the COVID ICU of a Tertiary Care Hospital in Assam During the First and Second Waves of the COVID-19 Pandemic

**DOI:** 10.7759/cureus.85090

**Published:** 2025-05-30

**Authors:** Dimpymoni Saikia, Basanta B Das, Pollov Borah, Bishnu R Das

**Affiliations:** 1 Community Medicine, Jorhat Medical College, Jorhat, IND; 2 Anesthesiology and Critical Care, Lakhimpur Medical College and Hospital, North Lakhimpur, IND

**Keywords:** coronavirus disease 2019, covid-19, covid-19 clinical presentation, covid-19 icu patients, covid-19 pandemic, demographic profile, first and second wave

## Abstract

Background: With the passage of time, COVID-19 strains have mutated, leading to newer strains, and reports are there on the differences in presentation, severity, and infectivity of COVID-19 in the first two waves of the pandemic.

Objective: Comparison of the clinico-demographic profile and outcomes of patients admitted to the COVID ICU during the first and second waves.

Materials and methods: A hospital-based cross-sectional study was conducted for a period of six months among patients admitted to the COVID ICU of Jorhat Medical College and Hospital, a tertiary care hospital in Assam, including 284 patients. Data were collected from bed head tickets available in the Medical Records Department office. The t-test and χ2 test were used to compare data between the two waves.

Results: In the first wave, most of the patients were 60 years and above, while in the second wave, the majority of cases were between 40 and 60 years (χ^2 ^= 13.002, *p *= 0.011). The presenting symptoms and the type of co-morbidities were similar in both waves. Mean delay in testing and hospitalization was significantly more in the second wave (*p *= 0.005 and 0.02, respectively). Requirement of high flow O_2_ therapy (*p *= 0.006) and ventilatory support (*p *= 0.03) was higher in the second wave. The treatment protocol remained the same in both waves, except for universal use of remdesivir and decreased plasma therapy use in the second wave. The mean duration of hospital stay was longer in the second wave (*p *= 0.02). There was a decrease in deaths from 42.1% to 36.1%, while discharges against medical advice increased from 2.1% to 12.5%.

Conclusions: The data of the study gave us an insight into the changes and similarities of the two waves. Understanding the disease at the local level can help us be better prepared in case of any further recurrence of the pandemic.

## Introduction

A series of severe respiratory disease cases of unknown origin were reported in Wuhan, Hubei province, China, in December 2019. The causative agent was later identified as a novel coronavirus (Sars-CoV-2), also referred to as "2019-nCoV" [1,2]. The disease was declared a pandemic by the WHO on 11th March 2020. As of 31st March, 2022, 225 countries and territories around the world were affected with over 487 million cases and 6 million deaths [3]. In India, the first case was reported on 30th January 2020 in Kerala. The first wave peaked around September 2020, with cases rising to 97894 (on 16th September 2020) [3].

On 14th December 2020, a new strain was reported from the UK, which was speculated to be 70% more transmissible than the old variant. Since then, several new strains and variants of importance have been identified [4]. The appearance of the new variant (SARS-CoV-2 VOC 2020 12/01) marked the beginning of the “second wave” in the UK that spread globally. The second wave gained momentum in India from March 2021 and lasted up to June 2021 [5].

The changes in trends of the first and second waves were studied and evaluated on an international and national basis. A study involving the COVID-19 patients of a tertiary care hospital in our locality will help us better understand the trends and changes the disease has undergone locally, helping us in any future crisis. The study primarily aimed to assess and compare the clinico-demographic profile and outcomes of patients admitted to the COVID intensive care unit (ICU) of Jorhat Medical College and Hospital during the first and second waves of the COVID-19 pandemic. The secondary objective was to determine the associated factors with the outcome of the disease.

## Materials and methods

Type of study

A hospital-based, cross-sectional, analytical study was conducted to fulfill the study objectives.

Study settings

The study was conducted at Jorhat Medical College and Hospital (JMCH) by using the bed tickets of the patients admitted to the COVID ICU of the hospital.

Study duration

The study was done for a period of six months from July 2021 to December 2021.

Sample size

The total COVID-19-positive cases admitted to the COVID ICU of JMCH during the two waves (from the start of the first wave till 30th June, 2021) were 949. Since the study population was less than 1000, to maintain the study's representativeness, data were collected from 30% of these patients [6].

**Figure 1:**
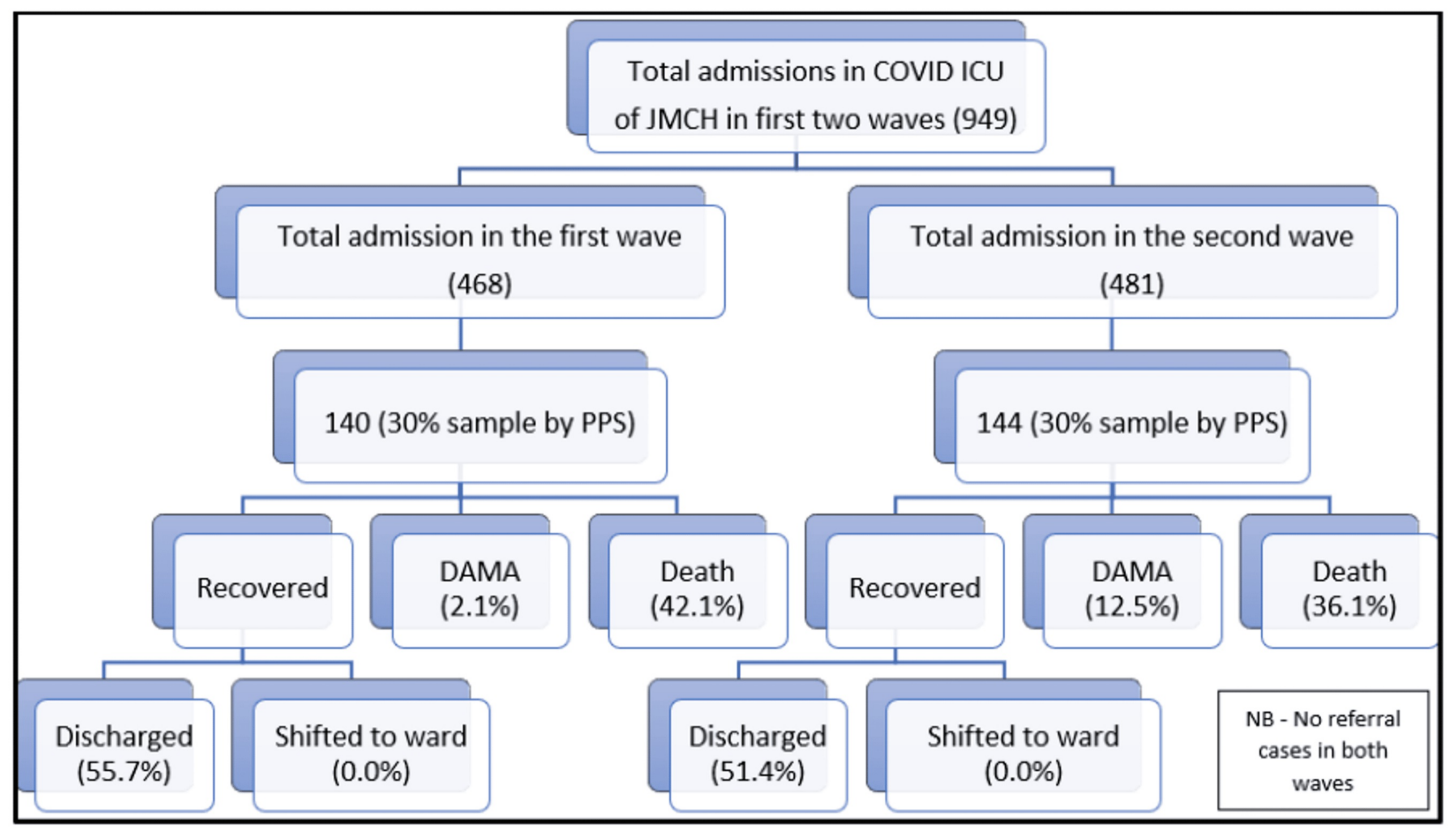
CONSORT diagram showing the sample selection and outcomes of the study participants admitted to the COVID ICU of JMCH. CONSORT: Consolidated Standards of Reporting Trials; JMCH: Jorhat Medical College and Hospital; PPS: probability proportional to size; DAMA: discharge against medical advice.

The first patient was admitted to the COVID ICU of JMCH on 7th August 2020, so starting from that date, data were collected till 28th February 2021 for analyzing the first wave. The second wave of the COVID-19 pandemic was considered from 1st March 2021 till 30th June 2021 as per Indian statistical data [5,7]. The total number of COVID ICU admissions from 7th August 2020 till 28th February 2021 (first wave) was 468, and from March 2021 till 30th June 2021 (second wave) was 481. Using probability proportional to size (PPS), the sample included from the two waves was as follows: first wave = 30% of 468 = 140; second wave = 30% of 481 = 144; the total sample size = 140 + 144 = 284. The sample selection is elaborated in Figure 1 below.

Inclusion criterion

All the bed head tickets of COVID-positive patients admitted to the COVID ICU of our tertiary care hospital were included.

Exclusion criterion

The tickets of the patients who were found to be incomplete or with missing pages were not included in the study.

Study method

Permission to pursue the study from the records of the Medical Records Department (MRD) was obtained from the principal of our institute. We had to first visit the COVID ICU to calculate the total number of patients admitted there by manually going through the registers and calculating the sample size to be taken. From the registers of the COVID ICU, we obtained the list of patients admitted to the COVID ICU, and their MRD numbers were noted. From this list, the cases were selected by simple random sampling. After that, we visited the MRD office to collect data from the selected bed head tickets of patients. Tickets that were torn, damaged, or incomplete were not included. In such cases, we included the ticket of the patient admitted to the COVID ICU with the next nearest MRD number. Patients who were admitted to the COVID ICU multiple times were included in the study only once. Confidentiality regarding patient identity was maintained.

Data analysis

Data were represented in tables using frequency, percentages, mean, and standard deviation. The chi-square test and bivariate logistic regression with a 95% confidence interval were used to analyze the association. A t-test was done to see the difference in means. SPSS version 23 (IBM Corp., Armonk, NY) was used for data compilation and analysis.

Ethical consideration

Ethical clearance was obtained from the Institutional Ethics Committee (Human) of Jorhat Medical College and Hospital, Jorhat (Issue No.: SMEJ/JMCH/MEU/841/Pt-2/2011/4510; dated: 10 September 2021).

## Results

The study included 284 cases, among which 140 cases were from the first wave and 144 from the second wave of the COVID-19 pandemic. The majority of the cases were male, accounting for 70.4% (200) of the cases. There was a significant difference in the age distribution of the cases between the first and second waves (χ2 = 13.002, df = 4, *p* = 0.011). In the first wave, more cases were aged 60 years or above (63, 45.0%), while in the second wave, the majority of the cases were between 40 and 60 years of age (72, 50.0%), clearly indicating an age shift to a younger age group in the second wave. The mean age of the cases in the two waves was 54.1 years and 53.5 years, respectively. The differences in the demographic profile of the two waves are shown in Table 1.

**Table 1:** Socio-demographic profile of COVID-19 patients admitted to the COVID ICU during the first and second waves. * Christianity, Jainism, and Sikhism.

Socio-demographic variables	First wave (%)	Second wave (%)	Total (%)	χ2	p-value
Age of the cases (in years)					
0-20	4 (2.9)	5 (3.5)	9 (3.2)	13.002	0.011
20-40	31 (22.1)	22 (15.3)	53 (18.7)		
40-60	42 (30.0)	72 (50.0)	114 (40.1)		
60-80	55 (39.3)	37 (25.7)	92 (32.4)		
More than 80	8 (5.7)	8 (5.6)	16 (5.6)		
Sex					
Male	100 (71.4)	100 (69.4)	200 (70.4)	0.134	0.714
Female	40 (28.6)	44 (30.6)	84 (29.6)		
Religion					
Hinduism	125 (89.3)	132 (91.7)	257 (90.5)	2.222	0.329
Islam	14 (10.0)	9 (6.3)	23 (8.1)		
Others*	1 (0.7)	3 (2.1)	4 (1.4)		
Locality (address) where the cases resided					
Jorhat	94 (67.1)	89 (61.8)	183 (64.4)	0.882	0.348
Outside Jorhat	46 (32.9)	55 (38.2)	101 (35.6)		
Setting of the area in which the cases resided					
Urban	70 (50.0)	69 (47.9)	139 (48.9)	0.123	0.726
Rural	70 (50.0)	75 (52.1)	145 (51.1)		

Among the 284 cases, 7% (20) were referred from other facilities. Of the cases, 63.0% were direct ICU admissions, while the rest were shifted from the COVID ward. For the diagnosis of COVID-19 at the time of admission, reverse transcription polymerase chain reaction (RT-PCR) test was done in only nine (3.2%) cases; in all other cases, rapid antigen test (RAT) was used. However, during the hospital stay, RT-PCR, RAT, and predictive tests (D-dimer test, procalcitonin) were done in 100% of the cases. All the cases had one or more COVID-related symptoms at the time of admission, which included fever, respiratory difficulty, cough, diarrhea, etc., as shown in Table 2. A total of 205 (72.2%) cases had associated co-morbid conditions. Some of the patients presented with multiple co-morbidities. The most common co-morbidities included diabetes mellitus, hypertension, cardiac diseases, chronic renal diseases, chronic obstructive pulmonary disease (COPD), asthma, etc. However, the patients presented with similar co-morbidities in both waves (Table 2).

**Table 2:** Differences in clinical history of the cases in the first and second waves of the COVID-19 pandemic. * Yates correction. RAT: rapid antigen test; SPO2: oxygen saturation; COPD: chronic obstructive pulmonary disease.

Characteristics of clinical and treatment profiles		Overall (284)	First wave (140)	Second wave (144)	χ^2 ^test	p-value
Presenting symptoms (multiple response)	Fever	186 (65.5%)	87 (62.1%)	99 (68.8%)	0.133*	0.987
	Cough	42 (14.8%)	21 (15.0%)	21 (14.6%)		
	Respiratory difficulty	163 (57.4%)	80 (57.1%)	83 (57.6%)		
	Diarrhea	3 (1.1%)	2 (1.4%)	1 (0.7%)		
RAT test for confirmation of COVID-19		275 (96.8%)	136 (97.1%)	139 (96.5%)	0.002*	0.9
Presence of co-morbidities		205 (72.2%)	101 (72.1%)	104 (72.2%)	0	1
Type of co-morbidities (multiple responses)	Diabetes mellitus	109 (38.4%)	56 (40.0%)	53 (36.8%)	0.306	0.580
	Hypertension	104 (36.6%)	49 (35.0%)	55 (38.2%)	0.623	0.43
	Chronic renal disease	24 (8.5%)	9 (6.4%)	15 (10.4%)	1.459	0.226
	COPD/asthma	9 (3.2%)	3 (2.1%)	6 (4.2%)	0.403	0.526
	Cardiac disease	8 (2.8%)	4 (2.9%)	4 (2.8%)	0.101	0.751
	Others (cancer, liver disease, etc.)	7 (2.5%)	3 (2.1%)	4 (2.8%)	0.001	0.974
SPO_2_ < 95% at the time of admission		261 (91.9%)	131 ((93.6%)	130 (90.3%)	1.035	0.3

The result showed that 100% of the cases in the COVID ICU required oxygen therapy; however, an increase in the requirement of high flow oxygen therapy was seen in the second wave from 18.6% to 27.1%, whereas medium flow oxygen therapy was required more in the first wave (*p *= 0.006). There was an increased requirement for mechanical ventilatory support in the second wave (17.1% to 27.8%, *p* = 0.03). The result also showed a clear decrease in the use of plasma therapy in the second wave. The differences in the clinical history and treatment profile of COVID-19 patients in the two waves are shown in Tables 2, 3.

**Table 3:** Differences in treatment profiles and outcomes of the cases in the first and second waves of the COVID-19 pandemic. * Yates correction; ** Fisher's exact test. DAMA: discharge against medical advice.

Characteristics of treatment profiles			Overall (284)	First wave (140)	Second wave (144)	χ^2 ^test	p-value
Amount of O_2_ required	Low flow		59 (20.8%)	22 (15.7%)	37 (25.7%)	9.959	0.006
	Medium flow		160 (56.3%)	92 (65.7%)	68 (47.2%)		
	High flow		65 (22.9%)	26 (18.6%)	39 (27.1%)		
Mechanical ventilatory support required			64 (22.5%)	24 (17.1%)	40 (27.8%)	4.599	0.03
Remdesivir			279 (98.2%)	135 (96.4%)	144 (100.0%)	**	0.028
Low molecular weight heparin (LMWH)			284 (100.0%)	140 (100.0%)	144 (100.0%)	-	-
Corticosteroid (Dexona)			284 (100.0%)	140 (100.0%)	144 (100.0%)	-	-
Plasma therapy			58 (20.4%)	55 (39.3%)	3 (2.1%)	58.185*	0.0001
Ivermectin			277 (97.5%)	134 (95.7%)	143 (99.3%)	2.461	0.117
Doxycycline			282 (99.3%)	138 (98.6%)	144 (100.0%)	**	0.242
Azithromycin			281 (98.9%)	138 (98.6%)	143 (99.3%)	0.001	0.975
Zinc			284 (100.0%)	140 (100.0%)	144 (100.0%)	-	-
Multivitamin			284 (100.0%)	140 (100.0%)	144 (100.0%)	-	-
Calcium			284 (100.0%)	140 (100.0%)	144 (100.0%)	-	-
Vitamin C			284 (100.0%)	140 (100.0%)	144 (100.0%)	-	-
Outcome of the patient		Recovered and discharged	152 (53.5%)	78 (55.7%)	74 (51.4%)	0.051	0.821
		Death	111 (39.1%)	58 (42.1%)	52 (36.1%)		
		DAMA	21 (7.4%)	3 (2.1%)	18 (12.5%)	9.659*	0.002
Cause of death		COVID	70 (63.1%)	37 (63.8%)	33 (63.5%)	0.001	0.975
		Non-COVID	41 (39.9%)	21 (36.2%)	19 (36.5%)		

Comparison of the two waves using the t-test showed a significant difference in the health-seeking behavior of the cases (Table 4). The duration between the onset of COVID-19-related symptoms among the cases and the test done was significantly longer in the second wave (*p *= 0.005). A similar increase in the duration between the onset of symptoms to hospitalization was seen in the second wave (*p *= 0.02). The duration of hospital stay was significantly longer in the second wave (*p *= 0.02).

**Table 4:** Comparison of health-seeking behavior and hospital stay of the COVID-19 patients in the first and second waves.

Variables (in days)	Overall (Mean ± SD)	First wave (Mean ± SD)	Second wave (Mean ± SD)	t test	p-value
Delay between the onset of symptoms and the test done	2.04 ± 1.937	1.71 ± 1.664	2.35 ± 2.127	2.819	0.005
Delay between the onset of symptoms to hospitalization	2.18 ± 2.108	1.89 ± 2.101	2.46 ± 2.085	2.295	0.02
Total duration of hospitalization	7.67 ± 5.501	6.94 ± 5.811	8.37 ± 5.106	2.205	0.02
Total duration of ICU stay	5.76 ± 5.069	5.28 ± 5.406	6.23 ± 4.691	1.583	0.1

The outcomes of the patients showed a decrease in the death rate in the second wave, but it was not found to be statistically significant. However, a statistically significant (*p = *0.002) increase in discharge against medical advice (DAMA) rate was observed in the second wave (Figure 1 and Table 3). Altogether, there were 111 deaths in the two waves. Among them, 63.1% (37 in the first wave and 33 in the second wave, total = 70) were directly attributed to COVID-19 (COVID death), which was significantly higher than non-COVID deaths (χ2 = 0.007, *p* = 0.933). When comparing the two waves, the cause of death (COVID or non-COVID) showed a similar pattern among the cases (Table 3).

To assess the factors associated with the outcome of the patients (recovery or death), DAMA cases were excluded from further analysis. Age, medium and high flow oxygen requirement, need for ventilatory support, and duration of hospitalization were found to be independently associated with patient outcomes.

The association of factors with the outcomes of the patients is shown by the binary logistic regression analysis in Table 5.

**Table 5:** Binary logistic regression analysis for factors associated with the outcomes of patients.

Variables	Sig.	Adjusted OR (Exp(B))	95% CI for Exp(B)
Age (in years)	0.005	1.030	1.009 – 1.051
Sex (male)	0.750	0.900	0.469 – 1.725
Religion (Hindu)	0.954	1.032	0.355 – 3.001
Setting (urban)	0.383	0.760	0.411 – 1.407
Presence of co-morbidity	0.154	1.728	0.815 – 3.665
Oxygen requirement (medium to high flow)	0.014	2.854	1.238 – 6.579
Need for ventilatory support	<0.001	4.884	2.288 – 10.425
Delay between the onset of symptoms to hospitalization	0.740	1.027	0.878 – 1.202
Total duration of hospitalization	0.013	0.878	0.793 – 0.973
Total duration of ICU stay	0.124	0.468	0.178 – 1.230

## Discussion

In our study, there was a clear increase in the number of COVID ICU cases in the age group of 40-60 years from 30% in the first wave to 50.0% in the second wave, whereas there was a decrease in the number of cases above 60 years of age from 45% to 31.3%. However, the number of cases belonging to the younger age group (below 40 years) was lower in the second wave. These differences in age distribution of the disease were found to be statistically significant (*p *= 0.011). A comparative study done in Reus, Spain, by Iftimie et al. among the hospitalized COVID-19 cases also showed an increase in the number of cases belonging to the 40-49 years in the second wave [8]. But the increase in cases below 40 years of age, as evident from their study, was not present in our current study. This difference might be because the present study was conducted only among those admitted to the ICU.

The present study did not show any difference in other socio-demographic variables. There was an increase in the number of cases from the rural area in the second wave, though it had no statistical significance. Sahoo et al., in their article on urban to rural COVID-19 progression in India, show a clear increase in COVID-19 cases in the semi-urban and rural areas as large numbers of migrant workers returned to their homes due to reactive policy decisions. Assam, among others, was one of the most affected states during this scenario [9].

The most common presenting symptoms were fever, breathing difficulty, and cough, which were found to be similar in both waves. This finding was similar to that found in the study done by Iftimie et al. in Spain [8]. Higher incidence of gastrointestinal symptoms among the hospitalized COVID-19 cases, particularly in the second wave, was reported by other studies and reports [8,10]. However, in our present study, gastrointestinal symptoms (mainly diarrhea) were present in very few cases, and no difference was seen between the two waves. This is probably due to ignorance on the patients’ side to correlate the symptoms with COVID-19, seek healthcare, and remain undiagnosed, or such patients were not serious enough to be admitted to the ICU setup. The present study also did not show any difference in the presence of co-morbidities among the cases in the two waves, similar to that of the findings in the prospective study done by Iftimie et al. in Reus, Spain [11]. The study done in Babool, Iran by Jalali et al. showed a variability in the presentation of co-morbidities, with some of the diseases like hypertension and malignancies increasing while some other diseases like cardiovascular diseases and asthma decreasing in the second waves [10].

The present study showed that the requirement for medium flow oxygen therapy was higher in the first wave among the ICU cases, while high flow oxygen requirement increased in the second wave. Similarly, the requirement for mechanical ventilatory support was higher in the second wave. Jain et al., in their report on the differences between the first and second waves of COVID-19 in India, also showed that there was an increase in the requirement of both oxygen therapy and mechanical ventilatory support in the second wave [12].

The treatment protocol used to treat ICU cases has seen many changes with the inclusion and exclusion of various medications over a period of time [13-16]. The present study showed that drugs like zinc, vitamin C, calcium, and multivitamins were given to all the cases admitted to the COVID ICU in both waves. The use of remdesivir among the ICU cases was high (96.4% and 100% in the two waves, respectively), which was as per the All India Institute Of Medical Sciences (AIIMS)/Indian Council of Medical Research (ICMR) COVID-19 clinical guideline stating that remdesivir may be considered in patients with moderate to severe disease (requiring supplementary oxygen) [17]. The use of low molecular weight heparin (LMWH) and corticosteroids (dexamethasone) was 100% in both waves, in accordance with the protocol. In our study, there was a significant decrease in the use of plasma therapy in the second wave. In fact, convalescent plasma therapy was dropped from COVID-19 treatment by the ICMR in mid-May 2021 [14,15]. The use of ivermectin (97.5%), doxycycline (99.3%), and azithromycin (98.9%) in COVID ICU cases was almost universal in both waves. However, the use of these three drugs was removed from the treatment protocol on 5th January 2023 [16].

The duration between the onset of symptoms to both diagnosis and hospitalization was longer in the second wave. These delays in seeking healthcare might be attributed to a change in the attitude of the people toward the disease. Even the number of people seeking DAMA was higher in the second wave in our study. Hesitancy to seek care, unwillingness, and fear might be some factors contributing to the present findings despite better availability of drugs and affordability of diagnosis and treatment in the second wave [12]. At the same time, the duration of hospital and ICU stay increased in the second wave, even though we saw that more ICU cases sought to be discharged against medical advice in the midst of the treatment.

The change in outcome of the patients cannot be commented upon definitively because of the high rate of DAMA in the second wave, though a decrease in the number of deaths had been seen. Various studies had shown a decrease in crude mortality in the second wave [8,10,12]; however, a study in Maharashtra by Zirpe et al. among ICU patients showed an increased death rate in the second wave [18].

The outcomes of the patients were found to be independently associated with an increase in age, need for medium and high flow oxygen, and ventilatory support requirement. Longer duration of hospital stay was also an independent factor.

Limitations of the study

As it is a record-based study, our findings depend mainly on the completeness, accuracy, and readability of the tickets. The outcome of the DAMA cases could not be assessed as it was a record-based study.

## Conclusions

The results of the present study showed that there were some differences in the clinico-demographic profile of the COVID-19 ICU cases between the first two waves of the COVID-19 pandemic. The cases in the second wave were younger, more cases were from rural areas, and the hospital catered to more cases from outside the district in the second wave. The clinical presentation and presence of co-morbidities were similar in both waves. However, the requirement for high-flow oxygen therapy and mechanical ventilatory support systems was higher in the second wave, suggesting an increase in severity. There was a greater delay in testing and hospitalization in the second wave. DAMA rate was also found to be higher, indicating a change in the health-seeking behavior. The outcomes of the cases were found to be associated with age, oxygen requirement, need for ventilatory support, and duration of hospitalization.

Understanding the presenting clinical features, requirements of various treatment logistics, and health-seeking behavior of the patients, especially at the local level, can help in the case of any future pandemic. Constant vigilance with the utilization of available data to make policies might help us in such situations without economic and social disruptions.

## References

[1] Zhou P, Yang XL, Wang XG (2020). A pneumonia outbreak associated with a new coronavirus of probable bat origin. Nature.

[2] Wu F, Zhao S, Yu B (2020). A new coronavirus associated with human respiratory disease in China. Nature.

[3] (2022). Worldometer. Countries where COVID-19 has spread. https://www.worldometers.info/coronavirus/countries-where-coronavirus-has-spread/.

[4] (2022). WebMD. COVID variants. https://www.webmd.com/lung/coronavirus-strains.

[5] Asrani P, Eapen MS, Hassan MI, Sohal SS (2021). Implications of the second wave of COVID-19 in India. Lancet Respir Med.

[6] (2021). St. Olaf College. Sample size - Institutional effectiveness and assessment. https://wp.stolaf.edu/iea/sample-size/.

[7] (2021). Worldometer. India coronavirus statistics. https://www.worldometers.info/coronavirus/country/india/.

[8] Iftimie S, López-Azcona AF, Vallverdú I (2021). First and second waves of coronavirus disease-19: a comparative study in hospitalized patients in Reus, Spain. PLoS One.

[9] Sahoo PK, Biswal S, Kumar H, Powell M (2022). Urban to rural COVID-19 progression in India: the role of massive migration and the challenge to India's traditional labour force policies. Int J Health Plann Manage.

[10] Jalali SF, Ghassemzadeh M, Mouodi S, Javanian M, Akbari Kani M, Ghadimi R, Bijani A (2020). Epidemiologic comparison of the first and second waves of coronavirus disease in Babol, North of Iran. Caspian J Intern Med.

[11] Iftimie S, López-Azcona AF, Vicente-Miralles M (2020). Risk factors associated with mortality in hospitalized patients with SARS-CoV-2 infection. A prospective, longitudinal, unicenter study in Reus, Spain. PLoS One.

[12] Jain VK, Iyengar KP, Vaishya R (2021). Differences between first wave and second wave of COVID-19 in India. Diabetes Metab Syndr.

[13] Wu Y, Feng X, Gong M (2023). Evolution and major changes of the diagnosis and treatment protocol for COVID-19 patients in China 2020-2023. Health Care Sci.

[14] (2021). ICMR removes ‘plasma therapy’ from COVID-19 management protocols. https://science.thewire.in/health/icmr-removes-plasma-therapy-from-covid-19-management-protocols/.

[15] (2021). The Hindu. ICMR drops plasma therapy from COVID-19 treatment guidelines. https://www.thehindu.com/sci-tech/health/icmr-drops-plasma-therapy-from-covid-19-treatment-guidelines/article34582184.ece.

[16] (2023). Ministry of Health & Family Welfare, Government of India. Clinical guidance for management of adult COVID-19 patients - 2023. https://covid19dashboard.mohfw.gov.in/pdf/ClinicalGuidanceforManagementofAdultCOVID19Patientsupdatedason05thjan2023.pdf.

[17] (2023). Ministry of Health & Family Welfare, Government of India. Clinical guidance for management of adult COVID-19 patients - 2021. https://covid19dashboard.mohfw.gov.in/pdf/COVID19ClinicalManagementProtocolAlgorithmAdults19thMay2021.pdf.

[18] Zirpe KG, Dixit S, Kulkarni AP (2021). The second- vs first-wave COVID-19: more of the same or a lot worse? A comparison of mortality between the two waves in patients admitted to intensive care units in nine hospitals in western Maharashtra. Indian J Crit Care Med.

